# Patient-Reported Use of Personalized Video Recordings to Improve Neurosurgical Patient-Provider Communication

**DOI:** 10.7759/cureus.273

**Published:** 2015-06-02

**Authors:** Andrew J Meeusen, Randall Porter

**Affiliations:** 1 Division of Neurological Surgery, Barrow Neurological Institute

**Keywords:** video recording, doctor-patient communication, memory, recall, anxiety, medico-legal

## Abstract

Background: Providing patients with a video recording of their visit with a medical professional is a common-sense method for improving patient-provider communication.

Objective: To describe the patient and provider experiences to video recording clinical medical encounters and providing the patient with a copy of the video for informational purposes.

Methods: Since 2009, over 2,800 patients of eight different neurosurgeons chose to be video recorded during their encounter with the doctor and were provided access to the recording to watch over again as a way to recall what the doctor had said. The video system was set up as a handheld video camera, and video files were downloaded and made accessible to patients via a secure Internet patient portal. Between 2012 and 2014, patients who participated were surveyed regarding their use of the video and what was recorded on the video. The experience of the providers from a clinical and medico-legal standpoint was also reviewed.

Results: Three hundred and thirty-three responses to the survey were received (39.2% response rate). More than half of patients (N=333; 56.2%) watched their video more than once, and over two-thirds (N=333; 68.6%) shared their video with a family member, friend, or another physician. Patients self-reported improved memory after watching their videos (N=299; 73.6% could remember more) and 50.2% responded that having the video made them feel more “at ease” with their medical problem (N=299). Overall, 88.0% of respondents indicated that their video had been helpful to them, and 98.5% would recommend having future visits video recorded. No patient made a comment that the video was intrusive or had prevented them from being open with their doctor. Finally, in the high-risk specialty of neurosurgery, none of the 2,807 patients who have been recorded since 2009 have used a video in a medico-legal action.

Conclusions: Patient responses to the recording system and having a copy of their video were very positive. Most respondents indicated that they had improved memory as well as decreased anxiety about their neurosurgical problem. There have been no legal challenges to date from giving patients access to the video recording. Our results indicate that the benefits to patients for expanding the use of video in medicine may outweigh perceived risks to providers.

## Introduction

Good provider-patient communication is important to help all patients understand complex medical information and to become active participants in their own medical care. This is especially important when providing informed consent for procedures when providers must explain the diagnosis, treatment options and alternatives, risks and benefits, medications and their uses, follow-up, and long-term goals. Many prior studies have shown that patients’ abilities to recall medical information are often inhibited by a number of factors. Certain interventions for improving comprehension and recall of medical information have been successful. These include written materials, audio recordings, and generic video recordings about medical conditions, treatments, and discharge instructions. Despite the pervasiveness of video both within and outside of medical offices, few studies have attempted to assess the impact that video recording has had from the patient’s perspective. In the present study, we examine the results of a satisfaction survey given to patients who were filmed using a personalized video recording system known as The Medical Memory (TMM). Since 2009, more than 2,850 patient consultations and follow-up visits have been recorded using this system by the authors. Additionally, we discuss the experiences of the providers using the video recording system and the implications of expanding the use of video recording in medicine from a clinical and medico-legal standpoint.

### Patient memory for health information

It is well-established in the literature that patients’ memory for medical information extends to just a few topics. Patients typically forget most of what their doctors would deem “key” information within hours or days of their consultation visit [1]. In one early review, Ley (1966) found that 43% of patients interviewed could not recall their diagnosis during their first week in the hospital. As the number of medical information statements given to patients increased, patients' ability to remember everything decreased, from 81.8% for two statements to just 3.0% for seven or more statements [2]. In three separate studies conducted between 1965 and 1976 in which recall was measured between five minutes and two weeks after the consultation, patients recalled between 50% and 63% of the statements made by their doctor [3-5].

Recall tends to decrease when influenced by stressful or anxiety-inducing situations, such as discussion of cancer, the prospect of major surgery, or for certain types of information on which patients may place greater importance [2, 6-8]. Makaryus and Friedman (2005) found that less than one-third of patients could recall all of their medications at discharge from a hospital emergency department, less than one-half were able to state their diagnoses, and just 14% were aware of the side-effects of newly prescribed medications [9]. In the older adult population – projected to encompass approximately one in six patients by 2030 [10] – Bankoff and Sandberg (2012) reported just 15% of total items given were spontaneously recalled upon testing [11]. This improved to 59% in a cued recall test. In a study measuring recall in healthy volunteers by Fortun, et al. (2008), 60% of whom were medical students with presumably a greater ability to recall medical information, just 12% could name the three trial drugs presented and 20% could not remember a single potential risk of taking one of the medications [12].

These factors have implications for the quality of the decisions that patients are making in collaborative medicine and the ability of patients to understand what medical procedures they are consenting for. In 2007, the American Medical Association produced the Health Literacy Program video “Health literacy and patient safety: Help patients understand,” in which one patient described as a film producer had not been able to read the informed consent materials and recall the information discussed in a preoperative consult [13]. The patient signed a series of consent forms, but could not read or understand all of them, and did not realize she was consenting to a hysterectomy. It was not until after the surgery that she became aware of the procedure at her postoperative follow-up visit.

In this paper, we present the self-reported experience of patients who received a video recording of their visit with a neurosurgical provider at a major institution. Additionally, we report on the experiences of the neurosurgeons who used the video recording system within their clinics from the perspective of whether or not video recording in this manner is intrusive, causes medico-legal problems, and/or whether personalized videos provide a greater benefit than audio alone or generic non-personalized informational videos.

## Materials and methods

### Video system setup

Two different methods of video recording patients were used, as the technology that was being used by the third-party video recording company changed in early October 2014. Prior to that date, video recordings were made using standard Sony HMX-QF20BN handheld video cameras mounted on tripods and fitted with 32 GB standard SD cards. Videos were recorded by the office staff setting up the cameras for each clinic day, then returning the SD cards and cameras each night. Each patient was required to sign a paper copy of a HIPAA waiver allowing recording of the visit and a copy of the video recording company's terms and conditions before recording began. (Informed consent for the video recordings can be retrieved upon request by the editorial office.) The lead author (A.M.) was responsible for downloading each video and creating a patient account through an administrator portal page, then sending a username and password to each patient through the secure messaging system. Patients received an email telling them their video was ready to be watched, typically within 24 hours of their doctor’s visit, including instructions on how to access and watch the video.

After October 2014, the video company switched out the handheld cameras and tripods for Nexus 7 tablet computers loaded with a proprietary mobile app. This app allowed patients to input their own username and password (thereby improving the security of the system), increased the video upload and patient account creation time (from 24 hours to less than one hour), and created an electronic consent process for video recording (thereby eliminating the need for paper forms). Patients entered their email address and date of birth on the tablet, created a secure personal identification number (PIN) to be used as a unique first password, and then electronically signed the consent forms before the recording proceeded. After the visit, the video was encrypted, uploaded wirelessly to a cloud server, and the patient was sent a confirmation email for their account. Patients signed in with their PIN and then created a longer permanent password before having access to the video to watch as many times as they would like. Importantly, protected health information (PHI) is erased from the tablet itself after a successful upload so security and privacy are maintained in the event a device is stolen or lost.

### Patient satisfaction survey

Beginning in December 2012, satisfaction surveys were sent out to patient users of the video recording system. Using an online survey collection tool, patients who had used the service were contacted by email and asked to fill out a 15-question survey developed by author A.J.M one week following a visit with a neurosurgeon at which they had been video recorded, and responses were received between 0 and 14 days after sending the survey (mean 2.1 days). This patient opinion survey was originally conceived as a tool to improve the video recording system and for customer service purposes, and as such, it has not been tested for validity; therefore, causality for our outcomes cannot be scientifically substantiated.

The survey questions asked patients to describe what had been shown on their video, their opinions about video recording in general, whether or not they had shared the video with others (friends, family, or other physicians), and about the quality of the technical aspects of the recording (video quality and audio quality). In addition, four questions asked patients to self-report whether or not they remembered more information from their doctor’s visit, were more “at ease”, were less anxious about their treatment plan or diagnosis, and were more satisfied with the doctor after watching the video. Table 1 shows the list of survey questions asked. Respondent comments were coded into various categories by the senior author (A.J.M.), including technical issues, comments about recall or anxiety, and comments relating to overall satisfaction, the results of which are presented in the sections below.

**Table 1:** List of Survey Questions and Response Types

#	Question	Answer Type
1	Who is your doctor? (List of participating physicians in answer column.)	Single-choice
2	Since your doctor’s visit, how many times have you watched your video? (Single-choice answer)	Single-choice
3	What problems did you encounter in using The Medical Memory website to view your video? (Comment box)	Comment Box
4	How would you rate the sound quality of your video? (Scale from 1-5)	Scale from 1-5
5	How would you rate the video quality of your video? (Scale from 1-5)	Scale from 1-5
6	If your doctor showed your imaging (CT, MRI, X-rays, etc.) on your video, how helpful was this to you when you viewed your video? (Scale from 1-5)	Scale from 1-5
7	Thinking about your medical problem, do you feel more at ease, less at ease, or about the same since viewing your video? (More, less, about the same)	Single-choice
8	Thinking about your medical problem, do you feel more anxious, less anxious, or about the same since viewing your video? (More, less, about the same)	Single-choice
9	Thinking about everything your doctor said during your visit, do you remember more, remember less, or remember about the same amount after watching your video? (More, less, about the same)	Single-choice
10	Overall, do you feel that The Medical Memory video was helpful to you? (Yes/No plus comment box)	Yes/No plus Comment Box
11	Is there anything you wish you had seen on your Medical Memory video that was not there? (Comment box)	Comment Box
12	What did you like least about your video? (Comment box)	Comment Box
13	What did you like most about your video? (Comment box)	Comment Box
14	Would you recommend The Medical Memory to others who are going to see a doctor? (Yes/No plus comment box)	Yes/No plus Comment Box
15	Do you have any other comments about your Medical Memory video to share? (Comment box)	Comment Box

As of December 2014, 333 surveys had been collected from 850 surveys sent, producing a response rate of 39.2%. Thirty-four respondents stated that they had watched the video zero times and were excluded from further analysis. The reasons given for not watching the video included not receiving a password to access the video online, a problem understanding how to get to the website to log in, the patient waited too long and the video access expired, the video was not needed by the patient, and bad internet connection affecting playability of the video. The remaining 299 surveys were included in the review. One hundred and eighty-three respondents (61.2%) were female and 116 (38.8%) were male. This proportion closely reflects the demographic makeup of all TMM users, where approximately 64% are female and 36% are male. 

## Results

### Use of the video recording

**Figure 1:**
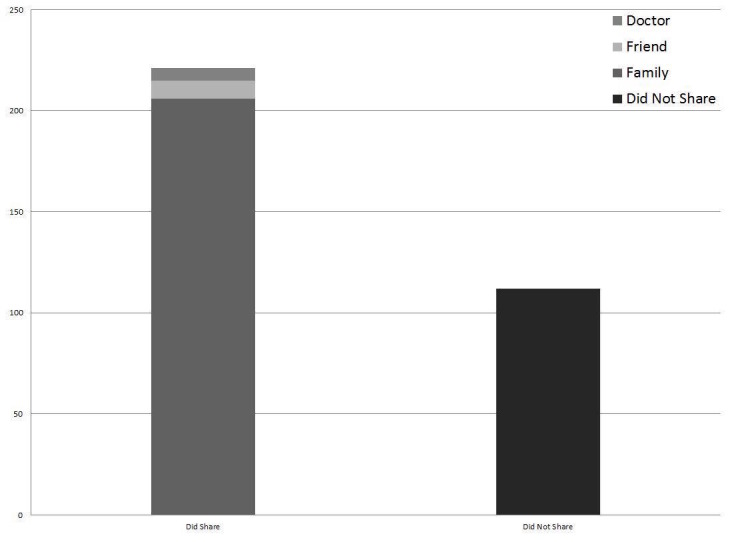
Sharing of Videos with Patient Family, Friends, and Other Physicians

Of the respondents, 131 (43.8%) watched their video exactly one time before taking the survey, and 168 (56.2%) watched their video more than one time, with six of these patients indicating that they had watched their video more than five times (mean: 3 views, range: 1-9 views). Two hundred and five respondents (68.6%) shared their video with a family member, 41 (13.7%) shared the video with a non-relative friend, and 13 patients (4.3%) shared their video with another physician, while 80 patients (26.8%) indicated that they did not show the video to anyone else (Figure 1).

Respondents indicated that their provider showed MRI, CT, x-ray, or other imaging studies in 143 videos (47.8%) and some kind of anatomical model in 90 videos (30.1%). Multinomial logistic regression analysis was performed to compare use of imaging in the video with overall satisfaction, and showed that respondents whose videos contained imaging or models were significantly more likely to feel “more at ease” (question 7), “less anxious” (question 8), “remember more” (question 9), rate the video as “helpful” to them (question 10), and answer "yes" to recommending the video to others (question 14) than respondents who did not indicate that imaging or models were shown (p<.001 for each question). One hundred percent of the patients who had been shown imaging on their video rated this as “helpful” to them, reflected in comments made about this aspect:

“I have [an issue] that the doctor found on my CT/MRI that I had no idea I had. I was seeing him for a different health issue. It’s been great to be able to show the video to family of my CT and MRI.”

“Seeing the MRI results let me see exactly where the problem area is and understand more of what the doctor was talking about.”

Overall, 263 patients (88.0%) indicated the video was helpful to them in some way, and 259 of these respondents (98.5%) indicated they would also recommend the video service to other patients. Four respondents who answered that the video was helpful to them stated that although they felt that the video had been helpful to them, they would not recommend the use of video to others, though their comments did not expound on why they felt this way.

### Recall and comprehension

**Figure 2:**
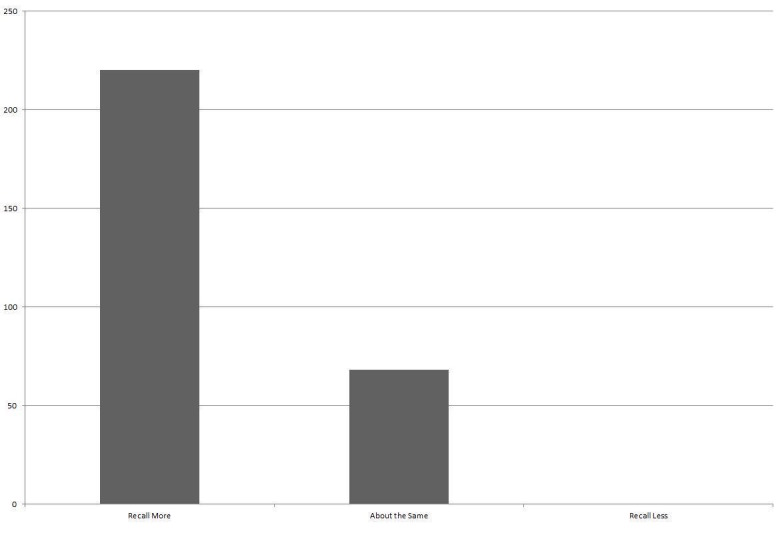
Patient Self-Reporting Their Ability to Recall What the Doctor Said No patient reported that viewing the video had a negative effect on their ability to recall information.

Many patients self-reported that having a video recording of their consultation to refer back to had allowed them remember what the doctor had told them better. Two hundred twenty respondents (73.6%) said they remembered more after viewing their video and 68 respondents (22.7%) remembered about the same amount, with no patient stating that they remembered less (Figure 2). Eleven patients did not respond to this question.

A total of 1,712 comments were received throughout the survey. Of these, 358 comments (20.9%) were in reference to the patient being able to remember something they had forgotten that the doctor had said or able to use the video to review the information. Some of the specific comments echoed quotes from other reference sources about medical information and recall:

 “Even though I researched my condition, I was caught off guard by the doctor’s response. There’s no way I would have remembered everything since we discussed several other possibilities.”

“The entire episode with the tumor was a blur to me… [on the video], information was revealed that I was unaware of because of my fogginess.”

“The conversation with the doctor brought out a point I would not have remembered if it were not mentioned on the video.”

Three comments (0.18%) made the complaint that the video was unnecessary because the patient had been able to remember everything in the visit without a video:

“Really, the video was not needed. My situation was not complicated at all, so there was nothing mentioned that was difficult to remember. However, the video would be extremely helpful if the situation were more complicated.”

Some patients also commented that having the video available saved them phone calls to the doctor’s office, which may help to reduce demands on staff or on the doctor themselves:

“Allowed me to review what was said during the meeting and saved a phone call to the doctor.”

“[I most liked] being able to go back and watch it, and when I had questions, they were in the video and I didn’t have to call the office.”

### Stress and anxiety

**Figure 3:**
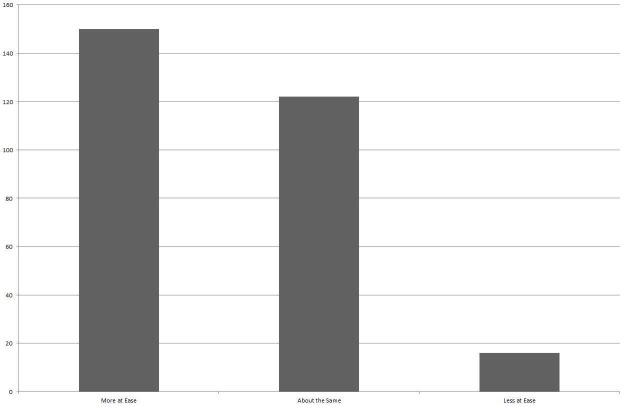
Patient Self-Reporting Their Comfort Level with Their Diagnosis and Treatment Plan

One hundred and fifty respondents (50.2%) indicated that having the video recording made them feel “more at ease” with their medical problem than before they saw their doctor compared with 16 (5.4%) who felt less at ease and 122 (40.8%) who felt about the same (Figure 3).

One hundred and eight respondents (36.1%) reported they felt less anxious about their problem compared with 23 (7.7%) who felt more anxious and 157 (52.5%) who felt about the same. Many of the comments received described experiences about how the video had helped the patient to become more comfortable with their diagnosis.

“The video helped me feel better about understanding my issue and [showed me] another way to solve my medical problem.”

“I was able to go over the info again at my own time when I was a little bit more at ease.”

“We get anxiety in new surroundings. [The video was helpful] so I could know exactly what was said and contemplate it later with no anxiety.”

Despite the fact that 23 respondents self-reported higher anxiety after watching the video, only two comments from the total 1,712 mentioned feelings of unease or discomfort:

“I really noticed how the doctor didn’t seem to listen to [me]…. I felt rushed in the exam room and watching it again didn’t help.”

“The resident told me surgery wouldn’t help me, but [the doctor] decided I need surgery. I’m not sure what to think. The video just stressed me out even more, I think.”

### Technical issues

While the majority of users indicated that they had no problems, 39 respondents did encounter some sort of issue and made a total of 103 comments about the problems they had encountered. These problems were coded into four categories: technical audio or video (problems involving the technology itself), password retrieval problems, camera user errors, and patient computer literacy issues. There was a total of 32 technical issues relating to extraordinarily long load times or audio/video sync issues (where the audio either lagged behind or was ahead of the video).

“The video freezing was VERY frustrating while watching it.”

“The audio and video were not in sync. The person I forwarded [the video to] experienced the same problem.”

These technical problems were mostly determined to be due to poor internet connections with the exception of one, where a patient had multiple videos which began automatically playing in sync with one another. This error was corrected with a software patch to the video playback system.

Eleven respondents had not received their password via secure email and had to call the office for assistance. Ten other respondents experienced a computer literacy problem, where they indicated it was difficult for them to figure out how to get to the video, even without any technical problems being present. In all of the following cases, the video was found to be loaded and working appropriately when the patient made contact to complain:

“I am computer handicapped. I had my son bring up and load to the computer.”

“My computer was not set up to view the program and I am very computer illiterate but finally got with the program!”

The largest number of issues involved errors being made by the doctors, resident, or medical assistants operating the video camera within the examination room. A total of 50 comments received from 37 of the 39 respondents who indicated a problem mentioned improper positioning of the camera, forgetting to turn the camera on or off at appropriate times, or the provider or medical assistant not positioning him- or herself in front of the camera appropriately. Prior to starting to video record patients, medical staff are given training by TMM personnel in proper setup and positioning of the cameras so as to capture the doctor-patient conversation appropriately, and a TMM support person is available to help with any questions which may arise during the first week of clinic for that office. Physicians are also asked to maintain some level of camera awareness when in the exam room, so that they can notice if they have moved out of frame during the course of an examination and adjust the equipment accordingly. Despite these attempts, not all videos have been perfect:

“The first 10 minutes was audio only; the video showed a blank white wall.”

"The doctor forgot to turn the camera on and had to repeat the visit.”

“There was a gap of a few minutes when the doctor had left the room to review my files. It would have been nice if he had turned the camera off during that time.”

### Video intrusiveness

In the present study, no patient made a comment that the video was intrusive or that they felt the video had made them feel uneasy or unable to discuss something with their doctor. Some comments mentioned that being on video actually helped them open up about their issue and make better medical decisions:

“Having the video allowed me to change my decision and change my surgery to include all three levels… per [my doctor’s] observations that I watched on the video.”

“Each time I re-watch the video I discover additional things. No way I could remember all this critical information without this video.”

## Discussion

### Previous interventions to improve patient recall

A major movement in the healthcare industry in 2014 focused on the concept of health literacy and making information conveyed by providers to patients more understandable by a larger proportion of the adult population, which may be responsible in part for improving patient recall of information. The typical focus of the health literacy movement is on designing new interventional methods of educating patients using written information (such as brochures or pamphlets written at a grade-level equivalent so as to be understandable by a majority of the population) or technologies, such as standardized or personalized audio recorded information and standardized or personalized video-recorded information. Improvements for recall and/or anxiety are shown throughout these different interventions from standardized audio-recorded information (net negative improvement) to standardized video education (low positive improvement) to personalized audio-recorded information (high positive improvement).

### Standardized audiotaped information

The use of standardized audiotaped information has not been closely studied, perhaps in part because it is inefficient to create standardized tapes when tape recording the consultation is just as convenient and provides information more appropriate to the patient’s specific issue. However, during our literature review, the authors found one study examining the effects of standardized audiotapes. Dunn, et al. found a linear increase in satisfaction scores between no tape (lowest), standardized audiotape information, and personalized consultation audiotaped information (highest) [14]. Dunn also found that while recall scores between the “no tape” and “consultation tape” groups were not significantly different, the provision of a standardized tape caused a significant decrease in recall scores over the consultation tape (29.8% versus 22.6%, P<.05).

### Standardized videotaped information

The use of standardized videotaped educational programs is a different intervention which has been shown to have positive effects, more so for recall and comprehension than anxiety. In a 2010 study by Armstrong, et al., the use of a video education program for informed consent and wound care information significantly increased intragroup knowledge scores as opposed to the control group, although no significant between-group knowledge score differences were found [15]. Atzema, et al. found similar results, that patients reviewing emergency department discharge information via an educational video were 3.5 times more likely to achieve a perfect knowledge score (three out of three questions about post-discharge care) than the control [16]. Standardized video discharge instructions were also measured by Bloch and Bloch (2013), who found that caregivers of children admitted to the emergency department showed improved recall and satisfaction when shown discharge information via video with written supplement versus video alone, both immediately after discharge (p<.0001) and one week after discharge (p<.0001) [17].

Informed consent is an aspect of medicine which necessitates strong participant recall and comprehension of information. Norris and Phillips (1990) conducted a study using video-recorded standardized informed consent information and found evidence that strongly supported the efficacy of a video for the informed consent process [18]. One hundred percent of the 200-patient cohort missed fewer than three questions on a recall questionnaire following the video informed consent as opposed to 30% of the control group who did not view the video. Ten percent of the control group also missed all ten recall questions.

In opposition to these findings, some studies have reported no significant differences in recall scores between groups using video education. Johnson, et al. found no differences between standard written information, standard information plus video, and standard information and video, plus formal nurse-taught education [19]. Bakker, et al. similarly reported no statistical difference between video education and nurse-taught education in oncology patients regarding chemotherapy [20]. Sonne, et al. also reported that no difference was found between video-assisted informed consent presented on an iPad and the control group, although over 97% of patients in the study self-reported that the video informed consent improved their comprehension [21].

### Personalized audiotaped information

Several studies have examined the use of consultation audio recordings to supplement recall or help reduce anxiety. Many studies agree that recall can be improved and anxiety can be reduced using audiotapes when patients are able to review them in a more comfortable setting or with a more comprehensive support structure of family or friends than is practical in a clinical office setting [7, 22-29].

Ong, et al. found audiotapes improved recall of information (p<.01), but that patients were also more satisfied with the information provided to them when they had access to the tape than without (p<.05) and 98% of patients believed that the audiotapes positively impacted their care [30]. Similar studies have reported that audiotapes improved patient recall of information [7, 15-18, 31-33]. One reason for this was described by Ford, et al., who studied the influence of audiotapes in participation in cancer consultations and found that simply knowing that the visit would be taped significantly increased the number of patients who asked for clarification on topics that they did not understand (p<.04) [23].

Bruera, et al. found that when personalized audiotapes were utilized in combination with written communications, recall of information significantly increased over the control group (88% versus 80%, P<.02) [34]. Additionally, a study by Cornbleet, et al. showed a significant improvement in information recall score in a small cohort (p<.0001) and a greater decrease in anxiety scores for the taped group over the non-taped group (p<.001) [35]. Tattersall and colleagues found no significant differences in recall between written and audio information in a series of studies between 1994 and 2002, although they did find benefits for audiotapes as both a patient information aid and a research tool [36-39].

### Personalized videotaped information

In 2004, Flory and Emanuel conducted a systematic review of interventions to improve recall for informed consent. They found that of twelve trials, which included video-recorded information, five trials documented improvements in either comprehension or recall of information, although they note, “that potential has usually not been realized in practice…. If this result were further verified, video technology might be a useful tool for improving retention of information” [24]. Schenker, et al. published a similar systematic review of 44 studies involving the effect of written, audio, or video interventions on recall, concluding that there is “fairly consistent evidence that additional audiovisual/multimedia programs… improve patient comprehension in informed consent, especially regarding risks and general knowledge about a procedure” [40]. Scott, et al. reported similar results in his systematic review of tape recordings versus written summaries; they found that four of the seven studies assessing recall showed improvements due to the tape intervention [41].

### Audio and/or video intrusiveness in the consultation 

The authors’ experiences with the initial reaction of a physician to offering audio or video recordings to their own patients has suggested that they think that the video camera may be intrusive or that the patient or the doctor would be unable to discuss the patient’s medical issues as fully as if they were not being recorded.

However, the literature on this subject seems to suggest the opposite. Martin and Martin determined that the majority of patients (50%) approved of videotaping in their consultation for teaching purposes, compared with just 11% who had reservations and 11% who disapproved [42]. Similarly, Campbell, et al. found that the vast majority of those filmed were not affected by the presence of the camera; in more than 95% of cases, the patient indicated they did not feel uneasy, and that they did not withhold any information from their doctor for fear that it might be on camera [43]. Campbell and colleagues found that 90% of their patients agreed to videotaping for teaching and training purposes without a corresponding drop in those patients’ satisfaction scores [44]. Butt, et al. obtained a 91% rate of patients who thought they were “helped or helped considerably” by the provision of an audiotape of their physician’s discussion and instructions and an 86% rate of patients who believed “that the taped communication improved their health care” [45].

Another way the video recording may be intrusive for providers is that it may slow down the clinical visit with patients. In an analysis of 339 videotaped consultations, half where the doctor was aware of being recorded and half where the doctor was unaware, Pringle, et al. discovered no significant differences between lengths of consults or the length of time taken on 26 of 27 common doctor and/or patient activities within the consultation; the one difference that was found to be significant was the time taken by the doctor to explore the patient’s problems [46]. Knowing they were on tape made doctors more likely to spend more time discussing this with their patients.

In our study, no patient made a comment that the video was intrusive or that they felt the video had made them feel uneasy or unable to discuss something with their doctor. Some comments mentioned that being on video actually helped them open up about their issue and make better medical decisions. In our experience, the video camera has not been intrusive for our physicians. When the doctor enters the consultation room, he pushes a single button to start the recording and then conducts his visit as normal. If using models or showing the patient their images, the doctor can choose to bring the camera with them to record that explanation, then replaces the camera back in the room. When finished, the doctor simply pushes the same button to stop the recording as he exits the room. While this represents a departure for doctors to their normal clinical routine when starting to incorporate video recording in their clinics for the first time, we have observed that they become adept at using the cameras within one to two weeks.

The fact that so many of our patients freely chose to have their visit video recorded and uploaded to the Internet, even paying a fee to use the service, speaks to the desire from the patient’s perspective that medical professionals begin to incorporate video recording technology into their practices. Video recording could be used as an adjunct to other patient empowerment and educational projects, such as OpenNotes (a patient-facing system allowing patients' access to their medical record and physician-produced written documentation) and multifunctional patient portal systems through practices’ existing electronic health record systems.

### Video vs. audio recording only

Another criticism of video systems is that audio recording is enough to capture the information that the doctor tells a patient. Indeed, sites like the Oliver Center in Texas are experimenting with providing audio recordings of individualized discharge instructions to patients for postoperative care [47]. However, verbal descriptions of important clinical information, such as a patient’s imaging studies or anatomical models, are not as effective as having the context of video associated with the doctor’s words. At our sites, the participating providers are encouraged to show and record explanations of the patient’s imaging studies and use models of the brain and spine to give the patient a visual of their problem. Of particular note, many of the patients who had not had their imaging studies shown and explained on the camera wished that had been included by their doctor (survey question 11):

“I would have preferred [the doctor] thought to bring the video camera to the x-ray when he gave an explanation, but it was too late to have it repeated or captured.”

“Having a discussion of my [problem] using the MRI and X-rays would have been helpful.”

“I would have liked to have [my doctor] demonstrate via a model or review of my x-rays taken prior to the appointment.”

“[I wish there was] more description using MRI and X-rays to help me get a better visual than just the doctor’s words.”

### Medico-legal concerns 

With the recent changes to the medico-legal infrastructure to tighten security for patient protected health information (PHI) through the Health Insurance Portability and Accountability Act (HIPAA) and the Health Information Technology for Economic and Clinical Health Act (HITECH Act), concerns have to be raised about the security of recording, uploading, and downloading recordings that contain PHI. Krackow, et al. queried six attorneys who perceived no increased risk of malpractice with the use of personalized audiotapes and suggested that there was no medico-legal basis for concern [48]. Krackow also found that it can be more common for a jury to believe a patient during a “he-said-she-said” lawsuit because from the patient’s perspective the visit is a unique event. By comparison, the physician gives similar advice or consents to multiple patients every day, and so a jury may believe that the physician would not remember information given to a patient during any one specific visit.

The authors contacted local malpractice carriers in Arizona to determine the medico-legal impact of providing patients with video recordings, one of whom stated that personalized video recording had the potential to be “the best evidence of the actual communication [between physician and patient]…. My guess is videotapes would be helpful to the defense in many instances on informed consent discussions, etc.” (Personal communication, Paul Giancola).

Another concern with video recording is whether the video is a part of the patient’s medical record. In the authors’ home state of Arizona, a medical record is defined as “all communications related to a patient’s physical or mental health or condition that are recorded in any form or medium and that are maintained for purposes of patient diagnosis or treatment” [49]. An internet search for other states’ definitions of “medical records” found variations on the details of the term, but all were thematically similar in that a medical record or health record was for the purpose of providing care, diagnosing, treating, or justifying payment for treatment of a patient [50-52]. In starting to offer video recording of patients, we sought counsel from the President of the American Health Lawyers Association, who indicated that if a video is being done to help the patient recall information and not for the care plan, “then the video is not maintained for purposes of patient diagnosis or treatment by a health care provider, then it is not part of the medical record” (Personal communication, Kristen Rosati).

The authors made their office the test site to evaluate a hypothesis that the use of personalized video recordings would not increase the legal risk in a high-risk specialty such as neurosurgery. Eleven physicians have provided almost 2,100 video recordings to patients since August 2012, and author R.W.P. has provided more than 1,500 of these videos since 2009 without a single legal challenge from a patient relating to or involving the video recording.

## Conclusions

Our survey showed that many patients self-reported increased ability to remember their doctors’ instructions and advice, and when they did not, many patients indicated that they knew that they could go back to the video recording to find information. Patients also self-reported decreased anxiety and a feeling that they were more “at ease” with their medical problem after reviewing the video, though it is unclear whether or not this is due to watching the video itself or to knowing that the information is readily available to them when needed. The majority of patients were pleased that videos were offered to them and would recommend having doctors’ visits recorded to other people. 

Despite having recorded more than 2,700 videos in a high-risk medical specialty, no medico-legal issues have ever occurred from either producing the video recording or from providing the video to a patient. It seems apparent from the results of our survey that patients not only want better, more easily understandable records of their doctor’s visits, but that the benefits of video recording seem to outweigh some of the legal risks that practitioners have cited in the past as a reason for not pursuing video records.

The authors recognize that there are limitations to the present study that may affect the ability of the data to be generalizable. First, we use custom-designed and non-validated surveys rather than peer-reviewed and validated outcome measures to assess anxiety, recall, and feelings about each patient’s experience with video recording their office visit. Second, this data describes patients in a single neurosurgical clinic only, and the authors expect to find different rates of video acceptance and outcomes in different types of practices and specialties. Future research may be warranted to determine how the uses of personalized video visits for other subspecialties or general practice differ from our experiences.
